# Perception and Experience Regarding Menopause among Menopaused Women Attending Teaching Hospitals in Erbil City

**DOI:** 10.5539/gjhs.v4n3p170

**Published:** 2012-05-01

**Authors:** Gazang Najmaddin Mustafa, Jwan Muhamad Sabir

**Affiliations:** 1 Nazdar Bamarny Family center, Erbil, Iraq; 2 Community Medicine Department, College of Medicine, Hawler Medical University, Erbil, Iraq

**Keywords:** menopause, age at menopause, perception, menopausal symptoms

## Abstract

**Background and Objectives:**

The timing of menopause, perception as well as menopausal symptoms varies between populations and within populations. The main objective of the present study was to assess women’s perception and experience regarding menopause, to find out symptoms and mean age of menopause and to study socio-demographic characteristics of menopaused women and to find out its relationship with their age at menopause and their knowledge about menopause.

**Methods:**

Over a period of eight months a descriptive cross sectional study were carried out at the outpatient departments of four teaching hospitals in Erbil city. A total of 500 menopaused women their age ranged from 40-60 years were interviewed using a close ended self administered questionnaire.

**Results:**

Mean age of menopause was 47.44 years with median age was 48 years, 4.4% had premature menopause and 23.6% had early menopause. The only factors that significantly associated with age at menopause were education and pattern of menstrual cessation and 93.4% of menopaused women were heard about menopause, 56.6% had prior knowledge of menopausal symptoms, cessation of menstruation was positive in 47.0% and 85.8% of women perceive menopause as natural condition and the most common menopausal symptoms were tiredness occurring in 83.2%.

**Conclusion:**

Most of menopaused women perceive menopause as natural condition and not aware about hormone replacement therapy and the mean age of menopause is comparable to that mean reported in other part of Iraq. Among menopaused women tiredness was the most common complaint was followed by hot flushes and night sweats.

## 1. Introduction

The term menopause means the end of the monthly menstrual cycle which is the central external marker of human female fertility and a natural menopause is deemed to have occurred after 6 months of secondary amenorrhea in a women aged 45 years or over (Edmonds et al, 2006). All women who live long enough will make transition to menopause (Wong et al, 2001). The menopause is based on the natural or surgical cessation of estrogen and progesterone production by the ovaries, which are a part of the body’s endocrine system of hormone production; in this case the hormones which make reproduction possible and can influence sexual behavior. Two hundred years ago only 30% of women lived through a menopause; now more than 90%will. Thus, the menopause transition and post menopause is very much a condition of the 20^th^ and 21^st^ centuries (Panay, 2007). As the world population increases and a large proportion of this population is made up of individuals older than 50, medical care specifically directed at post menopause women becomes an important aspect of modern medicine (Lobo, 2007). Age at which natural menopause occurs is between the ages of 45 and 55 for women worldwide. It is generally accepted that the average age at menopause is about 51 years in industrialized countries, but data are inconsistent for the developing world because of methodological problem (WHO, 1996). The most important factor determining a woman’s age at the menopause is the number of ovarian follicles (Block, 1952) and socio-demographic and behavioral characteristics found to have effect on determining the age at menopause (Ayatollahi et al, 2005).

Numerous factors including menopausal status, social background, and education, physical and emotional health may influence women’s knowledge and believes about menopause (Theisen et al, 1995; Avis and Mckinley, 1991). Attitudes, perceptions and expectations are part of the psychosocial phenomenon surrounding menopause (Avis, 1996) also there is an assumption in menopause research that attitudes to menopause are influenced by a range of cultural and social variables, which may in turn affect menopausal experience and symptom reporting (Ayers et al, 2010). A perception of the menopause as a positive event varies in different countries between 60%-90% (Leon et al, 2007; Kowalcek et al, 2005; Sallam et al, 2006) and menopausal symptoms are found to be less common in societies where menopause is viewed as positive rather than negative event.

The consequence of menopause are: (1) Immediate which involve: hot flushes, sweats, insomnia, anxiety, irritability, memory loss, tiredness, poor concentration, reduction in libido, (2) Intermediate: dyspareunia, dysuria, urgency, frequency, general aches, (3) Long term: osteoporosis, cardiovascular disease and dementia (Panay, 2007). Menopausal symptoms can really have an important impact in the daily, social and sexual life of postmenopausal women (Hassa et al, (2005).

Studies on women’s perception and experience regarding menopause and age at menopause are available from several Middle Eastern countries Age at menopause has been studied in Baghdad (Dhia Al-Deen and Sadik, 2009).

**Rational:** no similar study has been done in Kurdistan of Iraq especially in Erbil city regarding menopause.

### 1.1 Aim

This study was carried out to assess perception and experience regarding menopause among menopaused women and to evaluate their practicing at age of menopause in Erbil city.

## 2. Subject and Methods

A descriptive cross- sectional study was carried out over a period of eight months from1^st^ August 2010 to 1^st^ April 2011and this study was conducted at the outpatient department of the Maternity, Rizgary, Hawler and Raparin Teaching Hospitals. A convenience sample of 500 menopaused women of age 40-60 years was collected by direct interview and women who had a natural menopause included in this study. Women who underwent a hysterectomy or oophorectomy prior to their natural menopause and women who are complaining from symptoms due to medical diseases and past history of psychiatric disorder excluded from this study. A verbal consent was taken from each recruited respondent and informed that participation in the study was voluntary and permission was obtained from Directorate of Health and the four teaching hospitals in Erbil city to carry out the study.

A close ended self administered questionnaire was used to collect the information regarding socio demographic characteristics which include: name, age, residence, marital status, income, occupation, years of formal education and crowding index, menstrual and reproductive history with pattern of menstrual cessation, age of menopause (the age was directly recorded for those women who could remember the date correctly; women who could not remember were assisted by reference to dates of important events). In this study the age of menopause was grouped in to three classes <45years, 45-49years and >49 years (this classification depend on considering that early menopause less than 45 years (Panay, 2007) and premature menopause less than 40 years (WHO, 1996) then we arranged other two age groups to avoid recruited of all menopausal women in one age group to get good analysis data. Questions related to perception, practices, physical and psychological symptoms experienced by menopaused women were recorded.

### 2.1 Statistical Analysis

Questionnaires were coded after cleaning was done. Data were entered and analyzed using statistical packages for social sciences (SPSS, version 18). Two approaches were used; descriptive and analytic. The descriptive approach included calculation of the frequencies, percentages, mean SD.s and the second approach; t test was used to compare between means of two different samples, analysis of variance (ANOVA) that indicated by F sign was used to compare between more than two means. Chi-square test and Fisher’s exact test were used to test the association between categorical variables.

## 3. Results

In the study sample, age of menopause ranged from 37- 56 years with mean± S D of 47.44±4.35, median age of menopause was 48.00 years, 23.6% of women had menopause at the age of < 45 years, 40.4% between age 45-49 years and 36.0% at the age of > 49 years.

Age of women’s reported onset of menopause; 0.2% had their menopause at the age of 37 years, 0.4% had the menopause at the age of 56 years, 4.4% had premature menopause and 23.6% had early menopause.

As shown in Table 1, among 500 menopaused women who had natural menopause 458 from urban areas, 42 from rural areas. The highest age group of menopaused women were ≥ 55 years old with mean age was 53.6 years. Concerning the marital status most of menopaused women were married and the majority of menopaused women were housewives.

**Table 1:** Socio demographic characteristic of the study sample (n= 500)

Variable	No.	Variable	No.
Age of women (year)		Occupation	
40-44	11	Housewife	420
45-49	86	Manual partly skilled or unskilled	35
50-54	159	Non-manual skilled or semi skilled	41
≥ 55	244	Professional occupation	4

Residence		Years of formal education	
Rural	42	0*	355
Urban	458	1-6	71

Marital status		7-9	15
Single	4	10-12	11
Married	355	≥ 13	48
Widowed	139	Crowding index	
Divorced	2	<1.5	149

Income		1.5-2.9	234
Poor	60	≥3	117
Low	157		
Middle	272		
High	11		

* Illiterate and unschooled

As shown in Table 2, most of menopaused women heard about menopause and perceived menopause as a natural condition and the highest percentage of women said that their lives altered after cessation of menstruation.

**Table 2:** Distribution of women according to their perception (n= 500)

Perception	No.
Women had heard about menopause?	467
Previous knowledge of menopausal symptoms	283
Is it life altering?	356
Is it positive thing?	235
Is it natural or medical condition?	
Natural	429
Medical	71
Women must consult a physician?	377
Awareness about Hormone Replacement Therapy?	68
What foods are suitable for menopause women?	
Fruits and vegetables	191
Meats	6
Dairy products	54
Non specific foods	249

Table 3 shows practices related to menopause; half of women not consult a physician after menopause and most of them were not smokers with no significant association between smoking habit and age at menopause with P value > 0.05 as showmen in Table 4.

**Table 3:** Distribution of women according to practices

Practices	No.
Did she consult a physician? (after menopause)	245
Did she smoke?	
Smoker	76
Non smoker	412
Ex smoker	12
Did she discuss menopausal symptoms with doctors?	233
Did menopausal state affect on sexual desire?	173
Did she tell her husband about her menopause?	424

**Table 4:** Association of menopause age with smoking

Smoking	Total No	Mean ±SD of menopause age	menopause age group			

			<45 No(%)	45-49 No(%)	>49 No(%)	
Non smoker	412	47.44±4.35	99(24.0)	157(38.1)	156(37.9)	χ^2^=5.49, P=0.064
Smoker	76	47.30±4.33	17(22.4)	38(50.0)	21(27.6)	χ^2^=3.85, P=0.146
Ex- smoker	12	48.08±4.96	2(16.7)	7(58.3)	3(25.0)	NA*
Total	500	47.44±4.35	118(23.6)	202(40.4)	180(36.0)	
			F= 0.168, P= 0.845			

* Chi- square test not applicable

Table 5 shows that, tiredness was the most common complaint among physical symptoms and this complaint was followed by hot flushes and night sweats. About psychological symptoms, most common complaint was loss of short term memory followed by poor concentration.

**Table 5:** Physical and psychological symptoms experienced by menopaused women

Menopausal symptoms	No.
Tiredness	416
Hot flushes	357
Night sweats	345
Insomnia	232
Vaginal dryness	259
Dypareunnia	80
Urinary frequency	154
Dysuria	118
Urgency	111
Mood swings	207
Anxiety	214
Loss of short term memory	293
Poor concentration	269
Loss of self-confidence	20
Depressed mood	47

In this study although there was no significant variation in the mean age of menopause and years of formal education (F= 1.798, P= 0.128), there was a significant association between menopause age group and years of formal education (χ^2^=9.26, P= 0.009) with the highest percent of menopause age > 49 years was among those with ≥ 13 years of formal education and the lowest mean age of menopause and the highest percent 63.6% of early menopause was among those with 10-12 years of formal education as shown in Table 6.

**Table 6:** Association of menopause age with education

Years of formal education	Total No	Mean ±SD of menopause age	menopause age group			

			<45 No(%)	45-49 No(%)	>49 No(%)	
0	355	47.46±4.42	82(23.1)	150(42.3)	123(34.6)	χ^2^=1.79, P=0.407
1-6	71	47.28±4.37	22(31.0)	22(31.0)	27(38.0)	χ^2^=3.82 P=0.147
7-9	15	46.26±5.27	4(26.7)	5(33.3)	6(40.0)	χ^2^=0.32, P=0.851
10-12	11	45.00±3.68	7(63.6)	3(27.3)	1(9.1)	NA*
≥13	48	48.43±3.39	3(6.3)	22(45.8)	23(47.9)	χ^2^=9.26
Total	500	47.44±4.35	118(23.6)	202(40.4)	180(36.0)	
			F* * = 1.798, P= 0.128			

* Chi- square test not applicable

** F- ANOVA test was used to compare between means of menopausal age of different educational Levels. χ^2^ = was used to compare between the proportion of menopause according to age in each of educational level

Table 7 shows the variations in the mean age of menopause among women with different pattern of menstrual cessation were statistically significant (F= 20,950, P <0.001). The mean age was significantly lower among those with sudden cessation of menstruation than both gradual and recurrent bleeding (P <0.001). Age group at menopause was significantly associated with pattern of menstrual cessation (χ^2^=37.957, P <0.001) with the highest percent 40.3% of early menopause was detected in those with sudden cessation of menstruation with confidence interval regarding menopausal age group and pattern of menstrual cessation (44.63-46.22), (47.69-48.65), (47.35-48.98) respectively.

**Table 7:** Association of menopause age with pattern of menstrual cessation

Pattern of menstrual cessation	Total No	Mean ±SD of menopause age	menopause age group			CI

			<45 No(%)	45-49 No(%)	>49 No(%)	
Sudden (a)	134	45.43±4.64	54(40.3)	52(38.8)	28(20.9)	44.63/46.22
Gradual (b)	266	48.17±3.99	43(16.2)	118(44.4)	105(39.5)	47.69/48.65
Recurrent (c) bleeding	100	48.17±4.08	21(21.0)	32(32.0)	47(47.0)	47.35/48.98
Total a&(b,c) P <0.001	500	47.44±4.35	118(23.6)	202(40.4)	180(36.0)	
			F= 20.950, P <0.001 χ^2^=37.957, P <0.001			

*We not use Kruskall- Wallis test, we use ANOVA and χ^2^ for data analysis

Table 8 shows that knowledge about menopausal symptoms and awareness about hormone replacement therapy was significantly associated with residence (p= 0.011, p= 0.031 respectively) with higher proportion among urban women while no significant association was detected between hearing about menopause and residence (P= 0.345).

**Table 8:** Association between knowledge of menopaused women and residence

Residence	Women had heard about menopause No. (%)		Knowledge about menopausal symptoms No. (%)		Awareness about HRT No. (%)	

	Yes	No	Yes	No	Yes	No
Rural	41(97.6)	16(38.1)	1(2.4)	26(61.9)	1(2.4)	41(97.6)
Urban	426(93.0)	32(7.0)	267(58.3)	191(41.7)	67(14.6)	391(85.4)
Total	467(93.4)	33(6.6)	283(56.6)	217(43.4)	68(13.6)	432(86.4)
	P*= 0.345		χ^2^ = 6.39, P= 0.011		P*= 0.031	

* Fisher’s exact test

There was significant association between hearing about menopause (P= 0.031), menopausal symptoms (P <0.001) and HRT (P <0.001) with occupation with higher proportion among skilled and professional occupations as shown in Table 9.

**Table 9:** Association between knowledge of menopaused women and occupation

Residence	Women had heard about menopause No. (%)		Knowledge about menopausal symptoms No. (%)		Awareness about HRT No. (%)	

	Yes	No	Yes	No	Yes	No
Housewife	395(94.0)	25(5.9)	217(51.7)	203(48.3)	36(8.6)	384(91.4)
Unskilled*	29(82.8)	6(17.1)	24(68.6)	11(31.4)	8(22.9)	27(77.1)
Skilled**& Professional	43(95.5)	2(4.4)	42(93.3)	3(6.7)	24(53.3)	21(46.7)
Total	467(93.4)	33(6.6)	283(56.6)	217(43.4)	68(13.6)	432(86.4)
	χ^2^ = 6.940, P= 0.031		χ^2^ = 30.922, P <0.001		χ^2^ = 72.051, P <0.001	

* Manual partly Skilled or unskilled occupation

** Non-manual skilled or semi skilled occupation categorized with professional occupation for sake of statistical analysis

## 4. Discussion

The mean age at menopause in this study was 47.44±4.35 years with median age of 48. This result is more or less near to those reported in Baghdad (Dhia Al-Deen and Sadik, 2009), Northern Iran (Delavar and Hajiahmadi, 2011), Malay (Jahanfar et al, 2006) and Pakistan (Baig and Karim, 2006). The mean age at menopause in Asia is probably lower than in developed countries (Gold et al, 2001) and mean age of menopause in this study is lower than the mean age of menopause in similar studies in Poland (Kaczmarek, 2007) that reported the median age at natural menopause 51.2 years, USA (Gold et al, 2001) and Italian (Meschia et al, 2000) mean age at natural menopause were 51.4 years, 50.9 years respectively. Compared to the result of other studies in Asian and African countries (Jahanfar et al, 2006; Loutfy et al, 2006; Al-Sejari, 2005; Bairy et al, 2009) the result of this study is lower by one year. Possible explanation for this wide difference in menopausal ages may be related to the fact that ethnic, biological and cultural background may have an impact on age at menopause (Castelo-Branco, 2005; Ginsburg, 1991) or methodological differences between the present study and similar studies such as using retrospective recall during data collection.

In this study 93.4% of menopaused women heard about menopause and 56.6% had previous knowledge about symptoms this result is higher than the result of study conducted in Hyderabad Pakistan revealed that 78.79% of women heard about menopause but only 15.87% had knowledge about symptoms (Nusrat et al, 2008).

In the present study 71.2% of menopaused women stated that their lives altered after cessation of menstruation and perceiving menopause in a negative way in 53% which was similar to the result of a study conducted in Karachi Pakistan (Malik,2008) while 75% among Nigerian women (Ozumba et al, 2004) perceive menopause in a negative way. In another study, Thai women perceived menstruation to be an indicator of living and health (Punyahotra and Dennerstein, 1997) while respond to menopause in a negative way which may be attributed to the end of fertility, youth and creativity (Castelo-Branco, 2005).

In this study 85.8% of women perceived menopause as a natural condition which goes parallel with the perception of women in eastern societies who considered menopause as a natural process (Loutfy et al, 2006; Bairy et al, 2009; Blalt, 1953; Chang 1995; Adler, 2000).

Awareness and the use of hormone replacement therapy are generally low among populations from different Asian countries (Huang, 2010) in agreement with result of this study which revealed that 13.6% of women were aware about HRT.

More than two thirds of menopaused women 71.8% in a Danish study had discussed the menopause with a doctor; the more problematic the symptoms, the greater the likelihood that the women did consultation (Hvas et al, 2003) while in this study 49% of the women had consulted a physician and 46.6% discuss menopausal symptoms with the doctors, this may attributed to that women may not seek medical advice because they believe that menopause, like puberty, includes natural human changes that are part of development and ageing process (Wood & Mitchell, 1999).

In this study 82.4% of menopaused women were not smoker and we couldn’t find significant association between age at menopause and smoking habit, while a study in Egypt (Loutfy et al, 2006) reported higher percentage of non smoker; 98.4% and this attributed to the cultural differences .34.6% of menopaused women in this study mentioned that sexual desire decreased which could be attributed to that sexual life is a taboo topic that it is considered very private and may have effect on their answer whether affect or not and this result is not so differ with results of studies in Turkish population (Ayranci et al, 2010) and Karachi Pakistan (Ozumba et al, 2004) which reported 39.7%, 32.3% decrease sexual desire respectively.

In this study 84.8% of menopaused women told their husband about their cessation of menstruation and this may attributed to their perception about the menopause as a natural condition.

Results show that 51.8%, 16.0% of menopaused women complaining from vaginal dryness and dyspareunia respectively while a study among Saudi women reported lower percentage, 8.5% complaining from vaginal dryness and none of them experiencing dyspareunia (Al-Sejari, 2005) and 23.6% complaining from dysuria. While higher percentage of dysuria 35.1% reported in a study from Egypt (Loutfy et al, 2006).

Regarding psychological symptoms among the menopaused women in this study 58.6% with loss of short term memory which is inconsistent with the result of study done in Hyderabad Pakistan (Nusrat et al, 2008) revealed 62.10% with loss of short term memory and 9.4% of menopaused women in this study had depressed mood which is lower than that reported from study in Egypt which revealed that 60.7% were complained from this symptoms (Loutfy et al, 2006) probably this wide variation related to methodological differences and under estimation of depression in our community due to stigma and shame from mental disorders..

There was statistically significant association between age groups at menopause and education; highest percent of menopause age >49years was detected among those with ≥ 13 years of formal education and a study in Baghdad (Dhia & Sadik, 2009) also reveals significant association and this attributed to that educated women more likely better to deal with changes in menstrual period, consult doctors and receiving medications.

This study shows that sudden cessation of menstruation was significantly associated with earlier menopause than gradual cessation of menstruation and recurrent bleeding. This may attributed to that abrupt cessation may accompany sudden psychological trauma (pituitary shock) or may reflect abnormal or pathological ovarian failure that may occur at an earlier age (Vermeulen, 1993) and this finding coincides with the result of other studies in Baghdad (Dhia & Sadik, 2009) and Egypt (Hidayet et al, 1999).

Knowledge about menopausal symptoms and awareness of hormonal replacement therapy; the results show that there was significant association with residence, the highest percent of menopaused women from urban area had previous knowledge about menopausal symptoms and aware about HRT and this due to availability of different primary health care centers and consultation clinic with easier access to these health centers among urban peoples. Concerning occupation, highest percent of menopaused women with previous knowledge about menopausal symptoms and awareness about HRT were among skilled and professional occupation and this is in agreement with the result of a study in Egypt (Loutfy et al, 2006) and this attributed to that those who have skilled and professional occupation have better educational level and when they are complaining from any symptoms they consult a physician and getting more information.

## 5. Conclusion

Most of menopaused women perceive menopause as natural condition and not aware about hormone replacement therapy and the mean age of menopause is comparable to that mean reported in other part of Iraq. Among menopaused women tiredness was the most common complaint was followed by hot flushes and night sweats.
